# The Treatment of Tuberculosis

**Published:** 1918-11

**Authors:** 


***The Treatment of Tuberculosis.*** By Colonel G. E. Bushnell. *The Military Surgeon*, June 1918.

The author says that human beings generally possess a certain immunity against tuberculosis, this immunity being conferred to them by the natural absorption of small immunizing quantities of tubercle bacilli involving the development of appropriate antibodies in man's organism. Under some influences, as yet unknown, this immunity may accidentally disappear, thus allowing the infectious germs to be the strongest and to produce tuberculosis. The author insists, therefore, on the point that what must be sought first of all after the outset of the disease is the restoring of this complete immunity, rather than the destruction of the tubercle bacilli that seems impossible for the moment.

Anything that is for the general good of the patient, that increases the making of antibodies, that stimulates the nutritive activities of the cells of the body, is of benefit in helping to regain the lost immunity. This must constantly be kept in mind, and thus a rational treatment of tuberculosis will be easily understood and admitted by the patient.

In tuberculosis no medicines must be prescribed, simply a mode of life including the possibility of enjoying abundance of fresh air, rest, and good food well assimilated.

At the same time psychical treatment must interfere allowing no doubts and fears, and trying to strengthen constantly the patient's will.

*Rest.* Rest is absolutely necessary after the onset of tuberculosis. It has many good influences in abating the severity of the inflammatory process of the lungs, promoting cicatrization and encapsulation of the injured parts, and strengthening the body.

The more complete the rest the better the effects will be. Patients must practice lying perfectly still with relaxed muscles. Sitting requires constant contraction of certain muscles, and therefore must be considered as bad exercise. The patient must learn to control all his movements and also all nervous reactions. No excitement, no depressing emotions must be allowed; at first during relaxation periods, later at all times. He must be incited to suggest to himself sensations of comfort and well being. Psychical training given in this way proves very effective, but the treatment must not be interrupted one single day, on any excuse.

*Fresh air* is very important, but its prescription also must be dealt with carefully. A very ill patient does better in his room with windows opened, than lying out of doors and enduring bad weather. Covering should be rather light. Hot-water bottles should be used sparingly, warmth which leads to perspiration being bad.

*Exercise.* The tuberculous patient, whose temperature rises daily to 99.5° F., or upwards, should absolutely be kept in bed. As temperature falls, some exercise may be taken, if possible just after meals, in order to take advantage of movement as a stimulation to peristalsis. If small fluctuations of temperature occur it is impossible to give definitive rules for all cases, but if there is any doubt it is safer to prescribe rather too much than too little rest.

*Feeding.* Patients who are completely exhausted after suffering from deficient nourishment must first of all make up for this deficit. To those "over-feeding" is necessary; but to those only. For patients who have always had abundance of good food, stuffing with food can have only bad effects. The stomach and intestines must not be made to work too much in bad conditions, or else they will suffer rapidly.

*Symptomatic Treatment* is the treatment given to all patients in general. No special drugs are requested, unless perhaps cough medicines — in some cases, after hemoptysis, by example. Much can be done by teaching the patient to suppress useless cough. The belief that expectoration is infectious, and that it is very dangerous to swallow sputum, is quite a stupid error.

*Tuberculin.* The author disapproves of its use, as it may be very harmful, especially in advanced cases of tuberculosis. It seems to be most helpful only to those who need help least. It would be wise, therefore, to proscribe it severely, unless a very accurate diagnosis should prove its necessity.

